# Albumen in the Urine

**Published:** 1881-05

**Authors:** Chas. A. Doremus

**Affiliations:** Prof. Chemistry in University of Buffalo


### ALBUMEN IN THE URINE.*

BY CHAS. A. DOREMUS, M. D., PROF. CHEMISTRY IN UNIVERSITY OF BUFFALO.

Gentlemen—In selecting a theme for discussion at this, your annual meeting, my desire has been to have it combine, with theoretical interest, some practical points which alone you might consider worth your attention. Out of many topics, I have chosen one which will enable me to say a word in favor of a closer study of the department of medicine I am called to represent in your Alma Mater.

There is no part of chemistry, with which the medical student seeks to familiarize himself, more than with the composition of the urine, and in investigating that remarkable excretion, his

* Read before the Alumni Association, Feb., 1881.

chief endeavor seems to be in the direction of testing for its abnormal constituents; though from its practicability in the practice of medicine, this is laudable enough, yet by the superficial knowledge thus gained of the very essentials of chemical reactions, may fall into grievous errors, and are in special cases apt to fail completely in a correct diagnosis. The appearance of albumen in the urine under such varying circumstances, and from causes which may be totally opposed to each other, requires from the physician more than a passing notice and superficial testing, and this all the more since even as yet our knowledge of the varieties of albumen is so limited.

That we may understand the ground upon which we can build, with some show of its being a firm foundation, and separate that from other, about which there is still dissension, let us run quickly over the structure of the kidney, and some of the physical laws influencing the excretion of urine.

The examination of a vertical section of the kidney shows a most intricate system of tubes, some of which are arteries, others veins, and still others whose function is to bring the urine from the substance of the kidney to the pelvis of the organ. Under the microscope, these groups of vessels are found to ramify in numberless branches. These can best be described if we follow up the course of a urinary tube. Shortly after leaving the apex of a pyramid, it sends out branches in the direction of the cortical portion of the kidney. The branches run nearly parallel, however, and form bundles until they reach the cortical portion, where each branch winds off in a ribbon-shaped tube, and connects thus with a straight tube of small diameter. This is in its turn connected with a winding prolongation, termed the convoluted tube; not until it has, however, dipped down into the medulla and returned forming Henle's loop. The convoluted tube ends in a spherical, called the Malpighian body. This is the only portion of the urinary tube into which the blood vessels enter, but there the arteriole forms a network of capillaries, which, with the corresponding veins, forms a glomerulus. The veins indeed form a second plexus about the convoluted tubes, and a second system of arteries and veins is found about the lower portion of the urinary tubes.

The epithelial lining of the urinary tubes varies, each section having its special characters. We are enabled indeed at times to locate the seat of disturbance by the appearance of the epithelium cells in the urine.

The whole arrangement of blood vessels and tubes is such that some physiologists regard the kidney as a mere filter, and attribute the presence of albumen in the urine solely to influences such as pressure.

There is little doubt but that the excretion of the urine is mainly due to physical phenomena, yet we must also ascribe the presence of portions of the urine as being the result of chemical action.

The laws regulating transudation and osmosis are as yet imperfectly understood, and until we have increased knowledge upon these subjects, it will be difficult to explain all the conditions upon which the excretion of urine is dependent.

Transudation and osmosis differ in one very important particular, viz., that the latter does not depend upon the porosity of the separating medium, since a homogeneous septum can be used. The osmotic action is dependent upon molecular attractions and repulsions so nearly akin to chemical action as hardly to be distinguished from it. We should be prepared to find in the structure of the kidney the essential conditions, as far as membranes and fluids are concerned, for both transudation and osmosis.

Sections of the kidney show a different reaction with litmus, that portion which is richest in the straight tubes having an acid reaction. This is also in accordance with the facts ascertained by the deposition of carmine, injected into the blood and eliminated by the kidneys, in the epithelium of the latter; only where the reaction of the epithelium is alkaline, will the carmine produce a stain; the straight tubes may be full of carmine, yet their epithelium be unstained, because of its supposed acid reaction.*

The acid reaction of any portion of the lining membrane, even in the kidneys of animals voiding alkaline urines, points to a special function of the kidney more nearly allied to chemistry than to physics. The action of the kidney is undoubtedly dual, at least such must be the interpretation of the facts now known. The peculiar transformations it effects, of a chemical character, upon the constituents of the blood eliminated by the urine can only be explained by this hypothesis. Thus the influence of the kidney in the elimination of hippuric acid, hypoxanthine, coloring matters, &c.

Though the transudation theory of Ludwig accounts for a great many of the facts we know, regarding the excretion of the urine, it still leaves unexplained others of deep significance.

Thus it fails to show why the reaction of the urine is acid, the blood from which it is a filtrate being alkaline, nor does it explain altogether why the appearance of albumen is seen under abnormal circumstances only.

To be sure in most all cases of albuminuria the pressure in the blood vessels is increased, and the albumen would be pushed through under the circumstances, yet experiments, where albumen has been voided in the urine as the result of its injection into the blood, have failed to show increased pressure in the blood vessels.

It has been long known that albumen solutions may be filtered under pressure through membranes, but the percentage of albumen is always less in the filtrate than in the original liquid. The albumen in the urine is never more than 30 or 40 per cent., while that in the blood is 3—4 per cent. (Funke). Experiments made by Hugo Robbert (Jahresbericht über Thier Chemie, 1879,) show that the albumen in the urine comes from the glomeruli, though in pathological cases it may also transude from the tubules. His experiments did not exclude the possi-

*Kühner, Phys. Chan., p. 462.

bility of the albumen gaining access in the latter way, though it seems unlikely since albuminuria brought about in frogs and rabbits by injection of white of egg, occurs only through the glomeruli. The epithelium of the glomerulus becomes much changed in its form in albuminuria. (Kühne denies this). But whatever we may say in regard to the presence of serum albumen in the urine being due to increased pressure, what shall we say of the presence of other forms of albumen, as mucin, globulin, peptone, &c.

Harley (The Urine and its Derangements, p. 262), confirms Gigon's statements as to the presence of a form of albumen in normal urine. This form is not coagulated by heat or nitric acid, but precipitates under the action of chloroform and absolute alcohol.

Maixner (Jahresbericht über Thier Chemie, 1879, p. 351,) reports examinations of urine in which peptone was present.

Peptone was not found either in albuminuria, or albuminuria connected with renal disease; nor, as a rule, in general disorders or acute infectious disease, though in one case of typhus, one of carcinoma of the stomach and intestinal catarrh, and two of acute phosphorus poisoning. It was, however, found as a constant constituent of the urine in all diseases connected with suppuration, especially when the purulent exudation was great. Thus in plural and peritoneal exudations, congestive abscesses, bronchoblennorrhœa, &c. Peptone was also found constantly in cases of croupous pneumonia during the regenerative stage.

This form of albuminuria was called by Gerhard "Peptonuria," and was observed in cases of diphtheria, typhus, pneumonia, and phosphorus poisoning.

In opposition to Maixner's statement, however, Senator found peptone-like substances in cases of disease of the kidney, and Peter, in 28 cases out of 41 of albuminuria, Pavy, Schultzen, and Reiss also report cases.

In some cases of albuminuria, on dilution with water, a turpidity forms in the urine. This may be increased to a precipitate by carbonic acid.

Edlefson found paraglobulin in 31 cases of albuminuria. The reaction is more perfect in urines, rich in albumen. Senator and Peter confirm these statements. The former found the globulin to be especially abundant in cases of amyloid kidney. Next to this is acute nephritis. In five cases of catarrh of the bladder, fibrinoplastic substance was so abundant that it gave a thick clot with pericardial fluid.

Coagulable urines, or chylous urines are produced, when the globulin is increased. These cases are infrequent except in the tropics.

Other forms of albumen may at times occur; thus the white of eggs, as in disordered digestion, or after injection into the blood. We have said enough, however, regarding these points; my object being to put you on your guard, relative to the presence of albumen under various circumstances.

Recent investigations show that Graham's researches on the chemical action, during osmosis, and the separation of substances from each other, though held in chemical union, by simply passing through a membrane, will have great influence in elucidating many of the complex problems of assimilation, secretion and excretion. Kossel (Zeitschrift für Phys. Chem., p. 158, 1879) has shown by experiments that compounds, such as ferric acetate, or chloride-aluminium salts, &c., are resolved into different portions, some more diffusible than others, the diffusate often having an acid reaction. Even sodium phosphate is so resolved, and likewise the compound of carbonate of soda and albumen.

Graham found the diffusibility of substances through membranes or their osmotic power proportionate to the facility with which they decomposed. (Watt's Dict., p. 723, vol. iii.).

Kosell speaks very decidedly against the prevalent opinion of the "purification" of substances by dialysis.

From investigations as to the conditions under which albumen in any and all forms finds its way into the urine, we shall be led to a better understanding of the various types of the diseases in which it makes its appearance not only, but to a better idea of the physical and chemical laws regulating the functions of the kidney in health. From an increased knowledge in this direction, deductions can and will be made to explain similar functions of other organs.

NOTE.—A specimen of urine from a patient suffering from typhoid-pneumonia, though it gave no reaction for albumen, either with heat and nitric acid, or potassium terro-cyanide with acetic acid, yielded a goodly percipitate of peptone with alcohol. It is my intention to follow the matters up in other diseases.

